# Prevalence of Tongue Lesions in the Namakkal and Erode Districts: A Prospective Study

**DOI:** 10.7759/cureus.112934

**Published:** 2026-07-18

**Authors:** Deepakraju Krishnamoorthy, Suman Jhansi Lakshmi, Senthil Kumar B, Anand Kumar Jayaraj, Gomathi R, Keerthana Selvam, Sam Ponraj Davidson Sunder Singh

**Affiliations:** 1 Oral Medicine and Radiology, KSR Institute of Dental Science and Research, Namakkal, IND

**Keywords:** fissured tongue, geographic tongue, glossitis, precancerous conditions, tongue diseases

## Abstract

Background

Lesions of the tongue are markers of several systemic diseases and mirror the general health. In the Namakkal and Erode districts of Tamil Nadu - regions with high tobacco/areca nut usage and limited oral health infrastructure - prevalence data on tongue lesions remain sparse.

Aim

This study aimed to find out how frequently common tongue lesions occur in patients from these areas.

Methods

A total of 1,250 patients were examined, of which 880 presented with various tongue lesions. Demographic details and clinical examination findings of the lesions were recorded in a spreadsheet and subsequently analyzed using SPSS version 26.0 (IBM Corp., Armonk, NY, USA).

Results

The study population predominantly comprised middle-aged adults (31-50 years), with a male predominance (53.2%). Geographic tongue (32.5%) and fissured tongue (24.3%) were the major benign tongue lesions observed among others. Geographic tongue was almost equally prevalent in males (31.6%) and females (33.5%). Fissured tongue was more common in males (26.1%). Anemic glossitis was notably higher in females (18.0%) compared to males (8.1%). The prevalence of potentially malignant disorders in the study population was relatively low, affecting 8.2% (n = 72) of participants.

Conclusion

The result of the present study revealed that the majority of the tongue lesions were benign rather than malignant. Despite the limitations, this study provided essential data from the Namakkal and Erode districts of Tamil Nadu outpatients. Age-, gender-, and habit-stratified results emphasized the necessity of tailormade approaches for the integration of educational, preventive, and treatment-oriented approaches into public health campaigns to reduce progression risks in high-vulnerability cohorts.

## Introduction

The tongue is an anatomically complex muscular organ that plays a pivotal role in taste perception, mastication, deglutition, phonation, and orofacial development. Beyond its functional importance, the tongue serves as a clinically accessible window to both oral and systemic health. Alterations in its morphology, color, surface texture, and coating can reflect a wide range of local and systemic pathological processes, including nutritional deficiencies, hematological disorders, immunological conditions, infectious diseases, and potentially malignant disorders (PMDs) [1,2]. A contemporary review of oral-systemic connections emphasizes that tongue abnormalities represent one of the most diagnostically informative findings available to oral healthcare providers and may constitute the first, or sometimes only, visible manifestation of an underlying systemic disease [3]. This diagnostic utility underscores the importance of incorporating a systematic tongue examination into every routine dental and medical clinical encounter.

Tongue lesions constitute a significant proportion of oral mucosal lesions encountered in clinical practice worldwide. Epidemiological data indicate that their prevalence ranges from 2.39% to 15.1% in general population studies and up to 52.2% in academic dental institution outpatient settings, with the wide variation attributed to differences in sample size, geographic location, ethnicity, diagnostic criteria, and study methodology [4]. Among the most consistently documented tongue lesions across populations are geographic tongue (benign migratory glossitis), fissured tongue, coated tongue, and atrophic glossitis [4,5]. Less common but clinically critical findings include oral lichen planus, oral submucous fibrosis (OSMF), and non-healing ulcers, which are classified as PMDs and carry a risk of malignant transformation [6].

Geographic tongue has been identified as a chronic, immune-mediated inflammatory condition with a complex polygenic etiology involving HLA-Cw6, HLA-DR5, and recently identified IL36RN gene mutations - factors shared with generalized pustular psoriasis - while fissured tongue is widely regarded as a benign normal variant whose prevalence increases with advancing age, with global rates ranging from 0.6% to 60% [7,8].

In the Indian context, tongue lesions have been documented across diverse institutional studies, with results varying considerably by region and study design. Patil et al. [7] reported a tongue lesion prevalence of 12.07% among 4,926 dental outpatients across India, with coated tongue the most frequent finding, while Maloth and Shrinivas [3] identified geographic tongue as the leading lesion in a South Indian cohort. A recent epidemiological study from a tertiary care center in South India by Piplani et al. [8] found tongue lesions in 66.5% of dental outpatients, underscoring the high clinical burden of these conditions in specialist dental settings. Despite this growing national evidence base, data from semi-urban and peri-rural regions with distinct sociodemographic profiles remain critically underrepresented in the literature. 

The state of Tamil Nadu bears a disproportionately high burden of oral cancer, with tobacco use, areca nut chewing, and alcohol consumption serving as the primary risk drivers, particularly in disadvantaged and agrarian communities with limited access to dental care [9]. Recognizing this burden, the National Health Mission (NHM) of Tamil Nadu launched a statewide Oral Precancer Screening Programme, supported through primary health centers and sub-district hospitals, to facilitate earlier detection and management of PMDs [10]. Nevertheless, despite these policy-level initiatives, robust oral health surveillance data disaggregated at the district level - particularly for peripheral districts such as Namakkal and Erode - remain sparse. Both districts are characterized by a predominantly agrarian, textile-industry, and small-trade workforce, with well-documented high rates of smokeless tobacco and areca nut use, moderate oral health literacy, and limited specialist dental infrastructure. These factors constitute an epidemiological context that is known to predispose populations to an increased burden of both benign tongue lesions and PMDs [9,11].

A critical gap exists in the literature; to date, no study has specifically characterized the spectrum, distribution, and clinico-demographic patterns of tongue lesions in the Namakkal and Erode districts of Tamil Nadu. This absence of regional baseline data impedes the design of locally appropriate preventive programs, evidence-based clinician training, and targeted public health interventions for this population. Available national and South Indian data cannot be extrapolated without reservation to this setting, given the unique ethnic composition, occupational exposures, dietary patterns, and habit profiles of the region.

The present prospective cross-sectional study was therefore designed to determine the prevalence and clinico-demographic distribution of tongue lesions among patients attending a dental outpatient department (OPD) in Namakkal district, with the aim of (i) generating district-level baseline epidemiological data on tongue lesion prevalence, (ii) identifying age- and sex-specific patterns of common tongue lesions, and (iii) estimating the burden of PMDs in this population.

## Materials and methods

The present study was conducted in the Department of Oral Medicine and Radiology, KSR Institute of Dental Science and Research, Namakkal district, Tamil Nadu, India. Ethical clearance from the Institutional Review Board and Ethical Committee was obtained (IEC ref no: 390/KSRIDSR/IC/2023), and the study spanned a duration of two years (August 2023 to August 2025).

A convenience sampling technique was employed to recruit eligible participants who met the inclusion criteria during the study period. The inclusion criteria comprised all patients visiting the OPD only with tongue lesions during the study period who were willing to participate. Patients with a history of surgical intervention of the tongue, such as glossectomy, and those unwilling to participate were excluded from the study.

A total of 1,250 patients visiting the OPD were subjected to examination. The nature and purpose of the study were explained to all participants, and informed consent was obtained prior to inclusion. Patient details were recorded in a structured proforma, and clinical photographs of tongue lesions were documented. The collected data were entered into a spreadsheet (Microsoft Excel 2007, Microsoft Office, Microsoft Corporation, Redmond, WA, USA) and subsequently analyzed using SPSS software version 26.0 (IBM Corp., Armonk, NY, USA).

## Results

The present study analyzed 1,250 patients, of whom 880 presented with various tongue lesions. Age distribution peaked in the 21- to 40-year age group (38.6% combined: 21-40, 41-60), followed by the age group of 21-40 years (17.5%); children ≤10 years and elderly >70 years were least represented (3.6% and 2.8%). Gender distribution showed a slight male predominance (53.2% male, 46.8% female). Geographically, most patients were from Namakkal (63.7%) versus Erode (36.3%). Overall, lesion distribution identified geographic tongue (benign migratory glossitis) as the most frequent diagnosis (32.5%, n=286), followed by fissured tongue (24.3%, n=214) and anemic/atrophic glossitis (12.7%, n=112). Ankyloglossia accounted for 9.5% (n=84); coated tongue, central papillary atrophy, lichen planus, candidiasis, and traumatic ulcers comprised smaller proportions (6.1%, 5.5%, 4.8%, 3.2%, and 0.9%, respectively). Sex-specific patterns showed geographic tongue prevalence similar in males and females (31.6% vs 33.5%); fissured tongue was more common in males (26.1% vs 22.3%), while anemic glossitis was notably higher in females (18.0% vs 8.1%). Age-wise, ankyloglossia predominated in those aged ≤20 years and was rare in those aged >60, geographic tongue clustered in those aged 21-60 years, and fissured tongue increased in older adults (41-60 and >60 years). PMDs comprised 8.2% (n=72) of cases, with lichen planus/lichenoid reactions being most frequent (4.8%), followed by OSMF (2.0%) and non-healing ulcers (1.4%). These findings highlight the predominance of benign migratory and fissured tongue conditions, with a modest burden of PMDs.

Table 1 presents the geographic distribution of patients. Of the 880 patients with tongue lesions, the majority were from Namakkal district (63.7%, n = 560), while the remaining patients were from Erode district (36.3%, n = 320). 

**Table 1:** Geographic distribution of patients (n = 880)

Place	Frequency (n)	Percentage (%)
Namakkal	560	63.7
Erode	320	36.3
Total	880	100.0

Table 2 presents the distribution of tongue lesions. The geographic tongue was the most prevalent lesion with a prevalence percentage of 32.5% (n = 286). This was followed by fissured tongue [24.3% (n = 214)]. Anemic or atrophic glossitis was observed in 12.7% (n = 112), while ankyloglossia was noted in 9.5% (n = 84). Coated tongue, central papillary atrophy, lichen planus, candidiasis, and traumatic ulcers were observed in 6.1%, 5.5%, 4.8%, 3.2%, and 0.9%, respectively. 

**Table 2:** Distribution of tongue lesions (clinical diagnosis) (n = 880) *Asterisk indicates rare tongue lesions.

Tongue Lesion	Frequency (n)	Percentage (%)
Geographic tongue / benign migratory glossitis	286	32.5
Fissured tongue	214	24.3
Anemic / atrophic glossitis	112	12.7
Ankyloglossia	84	9.5
Coated tongue	54	6.1
Central papillary atrophy	48	5.5
Lichen planus / lichenoid lesions	42	4.8
Candidiasis	28	3.2
Traumatic / aphthous ulcer	8	0.9
Other lesions*	4	0.5
Total	880	100.0

Table 3 presents the distribution of tongue lesions by sex. The geographic tongue was observed in 31.6% in males and 33.5% in females. Fissured tongue was more common in males, with a prevalence of 26.1%. Anemic glossitis was higher in females (18.0%) compared to males (8.1%). Ankyloglossia was slightly more frequent in males (11.1%) than females (7.8%). 

**Table 3:** Distribution of tongue lesions by sex (n = 880)

Lesion Type	Male, n (%)	Female, n (%)	Total
Geographic tongue	148 (31.6)	138 (33.5)	286
Fissured tongue	122 (26.1)	92 (22.3)	214
Anemic glossitis	38 (8.1)	74 (18.0)	112
Ankyloglossia	52 (11.1)	32 (7.8)	84
Others	108 (23.1)	76 (18.4)	184
Total	468	412	880

Table 4 represents the age-wise distribution of tongue lesions shows distinct patterns across different age groups. Ankyloglossia was predominantly observed in individuals aged ≤20 years and was almost absent in older adults (>60 years). Geographic tongue was most common in the age groups of 21-40 years and 41-60 years. Fissured tongue primarily affected those aged 41-60 years and >60 years. 

**Table 4:** Age-wise distribution of common tongue lesions (n = 880)

Lesion	≤20 years	21–40 years	41–60 years	>60 years	Total
Geographic tongue	48	104	92	42	286
Fissured tongue	12	44	86	72	214
Anemic glossitis	10	38	42	22	112
Ankyloglossia	56	24	4	0	84
Others	24	68	58	34	184
Total	150	278	282	170	880

## Discussion

The present study provides the first prospective epidemiological assessment of tongue lesions in the Namakkal and Erode districts of Tamil Nadu, filling an important gap in the regional oral health literature. Of the 1,250 patients examined, 880 (70.4%) were found to have one or more tongue lesions, a proportion substantially higher than that reported in earlier Indian studies, which found a prevalence between 12.07% and 52.2% in dental outpatient settings [7,8]. This elevated proportion likely reflects the comprehensive and systematic nature of the tongue examination protocol employed, the demographic profile of the study population (predominantly middle-aged adults with known high-risk habits), and the referral patterns of the tertiary dental institution in Namakkal district.

Age and sex distribution

The study population predominantly comprised middle-aged adults in the age range of 21-60 years, consistent with national reports by Maloth and Shrinivas [3] and Patil et al. [7], who similarly identified middle-aged adults as the highest-prevalence group for tongue lesions. Male predominance (53.2%) was observed in the present cohort, a pattern documented across multiple global studies including those from Libya [8], Jordan [9], Maharashtra [4], and Turkey [5]. This sex pattern has been widely attributed to higher rates of tobacco smoking, bidi use, and areca nut chewing among males in India [11]. Supporting this, a propensity score-matched case-control study by González-Álvarez and García-Pola identified male sex as a significant independent risk factor for tongue lesions after adjusting for age, tobacco, and alcohol consumption, underscoring that sex-specific hormonal and behavioral factors each contribute independently to tongue lesion risk [12].

Geographic tongue

Geographic tongue (benign migratory glossitis) was the most prevalent lesion, affecting 32.5% (n = 286) of patients with tongue lesions. This rate exceeds previously reported Indian figures - 24.3% by Maloth and Shrinivas [3] and 16.4% by Patil et al. [7] - and is higher than the global median but lower than the 51% reported by Shinde et al. [4] from Maharashtra. Globally, published prevalence of geographic tongue ranges from 0.1% to 14.3% in general population samples but is consistently higher (up to 32-51%) in dental outpatient cohorts [13]. The variation likely reflects geographic, genetic, and ethnic influences, as well as differences in clinical examination thoroughness. 

In the present study, geographic tongue showed a near-equal sex distribution, with a marginally greater female prevalence (33.5% vs. 31.6% in males), consistent with findings from a retrospective Polish cohort study by Ślebioda et al., which reported a female-to-male ratio of 52:48 among 100 patients with geographic tongue evaluated over a 10-year period [14]. Geographic tongue is recognized as a chronic, relapsing, immune-mediated inflammatory condition with a polygenic etiology; recent evidence has identified mutations in the IL36RN gene - shared with generalized pustular psoriasis - as well as associations with HLA-Cw6, HLA-DR5, and HLA-DRW6, suggesting overlapping immunological pathways with psoriasis [13,15]. A 2024 case-control study from West China Hospital further demonstrated a significant association between psoriasis and both geographic and fissured tongue in an Eastern Asian cohort, reinforcing the importance of screening psoriatic patients for oral tongue lesions [16]. Hormonal influences, atopy, and psychological stress have also been proposed as modulatory factors [15]. Geographic tongue was most prevalent in the age groups of 21-40 and 41-60 years (n = 104 and 92, respectively) in the present study, consistent with the adult predominance reported by Jainkittivong et al. [6] and Darwazeh et al. [9].

Fissured tongue

Fissured tongue was the second most prevalent lesion (24.3%, n = 214). The global prevalence of fissured tongue is highly variable, ranging from 0.6% in South Africa to 27.7% in Brazil, with recent Indian studies reporting between 13.0% and 28.6% [17,18]. The prevalence in the present study is comparable to Brazilian figures reported by Shareef & Ettefagh. [13] (27.3%) and to the 24.61% observed in patients over 60 years by Maloth and Shrinivas [3]. The proportion observed here is higher than the 13.51% reported in a recent Nepalese tertiary care center study by a descriptive cross-sectional design using similar convenience sampling, possibly reflecting differences in the age and habit composition of the studied populations [19].

A clear age gradient was observed for fissured tongue in the present study, with the greatest burden in individuals aged 41-60 years (n = 86) and >60 years (n = 72). Male predominance for fissured tongue (26.1% vs. 22.3%) was observed in the present cohort, consistent with studies by Piplani et al. [8] and NOHP Tamil Nadu entry [9]. This age-related increase is consistent with reports by Ehsan et al. [15] and Hu et al. [16] and supports the hypothesis that aging-associated comorbidities, including xerostomia, nutritional deficiencies, reduced salivary buffering capacity, and attenuated mucosal immune surveillance, cumulatively predispose the dorsal tongue to fissure development. Male predominance for fissured tongue (26.1% vs. 22.3%) was observed in the present cohort, consistent with studies by Piplani et al. [8] and NOHP Tamil Nadu entry [9], in which higher tobacco use and associated salivary gland dysfunction among males was the proposed mechanism.

Anemic (atrophic) glossitis

Anemic (atrophic) glossitis constituted 12.7% (n = 112) of all tongue lesion cases, with a marked and statistically notable female predominance (18.0% vs. 8.1% in males). This sex disparity is consistent with national epidemiological data: the National Family Health Survey-5 (NFHS-5, 2019-2021) documented that 57% of Indian women aged 15-49 years are anemic, with iron deficiency as the principal cause [20]. Atrophic glossitis, characterized by depapillation of the dorsal tongue surface, erythema, and burning sensation, is a recognized oral manifestation of iron, vitamin B12, and folate deficiency [21]. A comprehensive study of 1,064 atrophic glossitis patients by Boukssim and Chbicheb found significantly lower mean serum vitamin B12 and folic acid levels, and a higher frequency of serum iron deficiency in younger (≤50 years) compared to older patients, with female patients disproportionately affected [22]. A case series by Hill et al. [23] further confirmed that oral manifestations including glossitis, glossodynia, and recurrent ulcers may serve as early clinical indicators of systemic vitamin B12 deficiency and pernicious anemia. The high burden of anemic glossitis in females observed in the present study underscores the need for routine hematological referral when atrophic glossitis is detected, particularly in women of reproductive age presenting to dental OPDs in Namakkal and Erode districts.

Ankyloglossia

Ankyloglossia was observed in 9.5% (n = 84) of tongue lesion cases, with a predominant predilection for individuals aged ≤20 years (n = 56) and near-complete absence in those above 60 years. A slight male predominance (11.1% vs. 7.8%) was observed. Globally, the estimated prevalence of ankyloglossia ranges from 4% to 11% across populations, as summarized in a systematic review and meta-analysis by Hill et al. [23], which reported an overall prevalence of 8% (95% CI 6-10%) in children under one year. A 2022 meta-analysis using multiple assessment tools confirmed that reported prevalence varies substantially depending on the diagnostic criteria and classification system employed [23]. The proportion observed in the present study (9.5% of tongue lesion cases) is broadly consistent with global estimates when interpreted within the context of the younger patient subpopulation. The marked absence of ankyloglossia in adults over 60 years of age - consistent with findings by Koay et al. [24] (1.7% in Malaysian adults) and Vörös-Balog et al. [25] (0.88% in Hungarian children) - likely reflects prior frenectomy procedures and the age demographic of the institution's outpatient cohort. The functional consequences of ankyloglossia extend beyond breastfeeding difficulties, encompassing articulation disorders, impaired jaw development, and orofacial muscular imbalances, reinforcing the importance of early detection and timely interdisciplinary management [26].

Other tongue lesions

**Figure 1:**
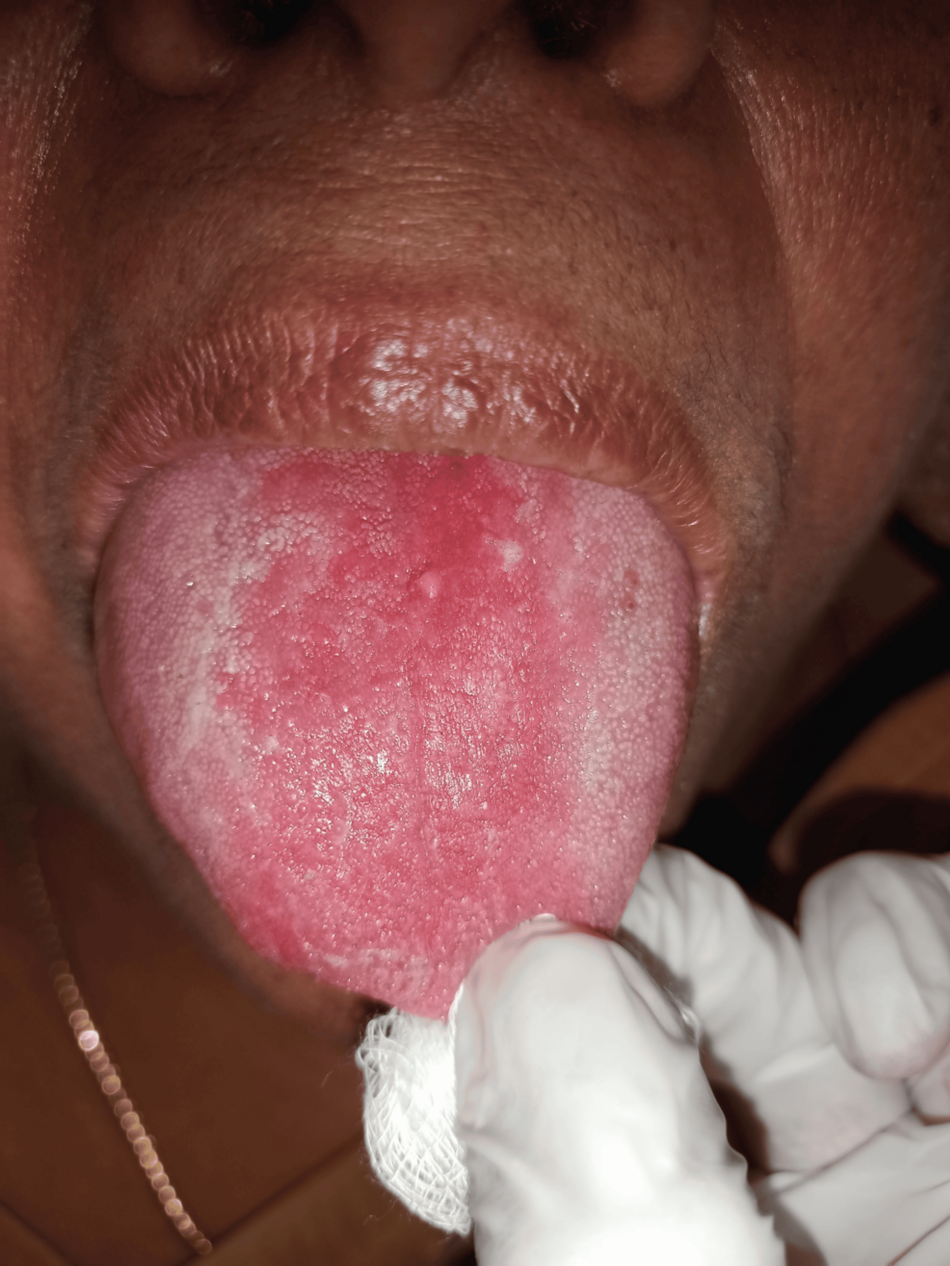
Central papillary atrophy

**Figure 2:**
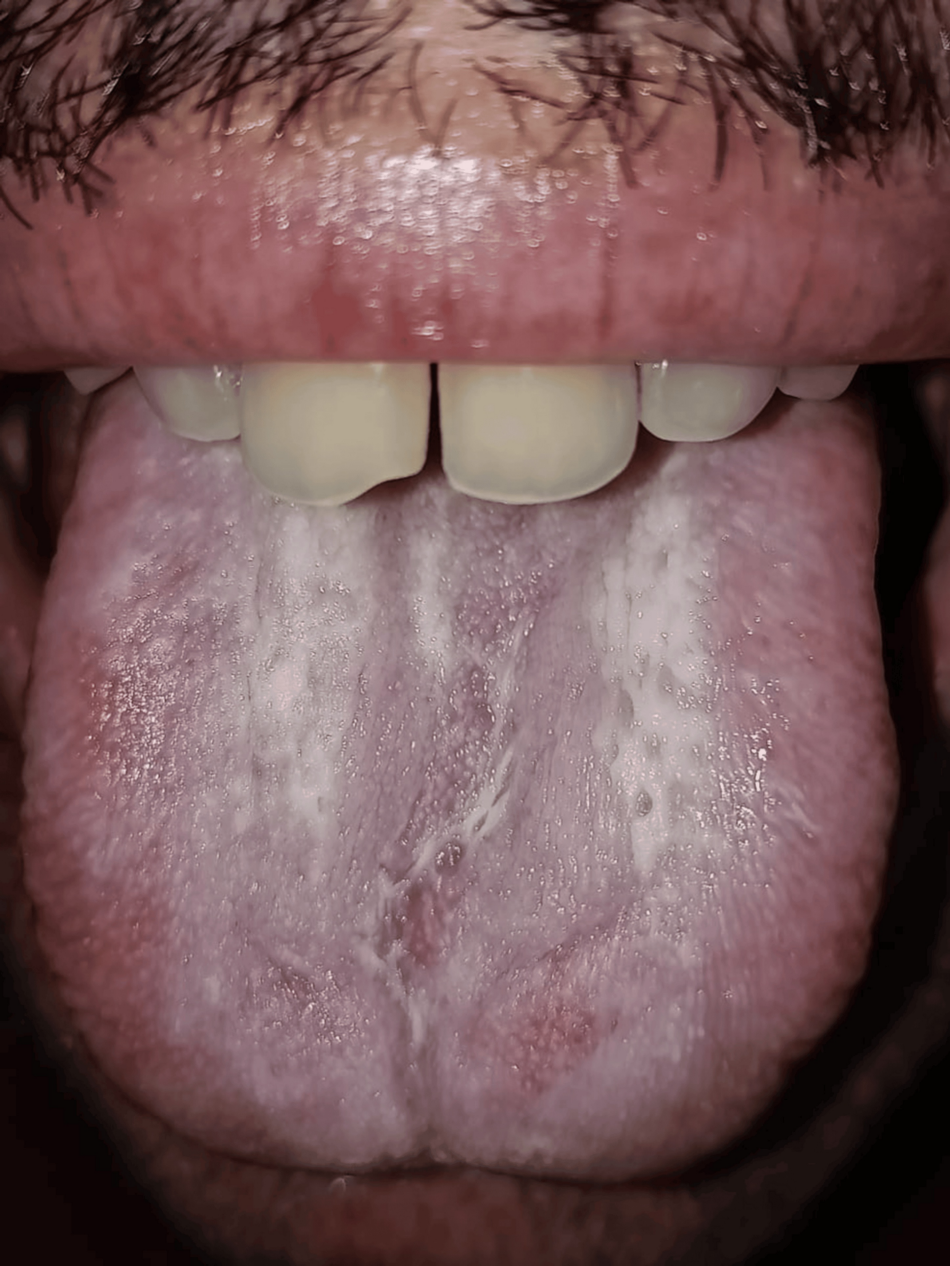
Candidiasis

Less common tongue lesions included coated tongue (6.1%), central papillary atrophy/median rhomboid glossitis (5.5%) (Figure 1), oral lichen planus/lichenoid reactions (4.8%), oral candidiasis (3.2%) (Figure 2), and traumatic/aphthous ulcers (0.9%). Central papillary atrophy of the tongue, often representing chronic erythematous candidiasis, may serve as an early clinical indicator of underlying immunosuppressive conditions such as diabetes mellitus or HIV infection, as highlighted in published literature on oral-systemic relationships [27]. The observed prevalence of oral candidiasis (3.2%) is relatively modest and may reflect the predominantly immunocompetent status of the patient cohort, as well as the absence of microbiological confirmation in the present study. The rarity of traumatic and aphthous ulcers (0.9%) is consistent with the outpatient context and the predominantly non-emergency referral pattern of the institution.

Potentially malignant disorders

PMDs were identified in 8.2% (n = 72) of patients with tongue lesions, with lichen planus/lichenoid reactions as the most prevalent (4.8%), followed by OSMF (2.0%) and non-healing ulcers (1.4%). This PMD burden is markedly higher than the 0.2% reported by Shinde et al. [4], though lower than the 13.7% reported from Madhya Pradesh, where OSMF was the dominant entity [6]. The predominance of lichen planus over OSMF in the present cohort contrasts with patterns seen in Northeast India, where gutkha and pan-masala use drives OSMF rates significantly higher. The Namakkal-Erode region's exposure profile - characterized by traditional tobacco forms and areca nut chewing without pan masala additives - may partly explain this difference. A 2025 meta-analysis by Sivaramakrishnan et al. confirmed that areca nut chewing is significantly associated with a higher OPMD risk in smokeless tobacco users [11]. A hospital-based study of tobacco users in Kolkata by Pandya et al. [27] reinforced the association of gutka and smokeless tobacco with leukoplakia, OSMF, and erosive lichen planus, with male predominance in habit-related PMDs. Given that the Global Burden of Disease Study 2021 projects a continued increase in lip and oral cavity cancer incidence globally, with South Asia identified as a high-burden region, and given Tamil Nadu's established NHM oral precancer screening programme [10], the present study's PMD data provide valuable institutional-level evidence to supplement and strengthen district-level surveillance efforts.

From a broader clinical perspective, the present study reinforces that tongue examination must be a non-negotiable component of every dental consultation, particularly in regions with high tobacco and areca nut use. The dual burden of benign symptomatic lesions and PMDs in the same outpatient cohort supports an integrated approach to tongue health: while most lesions require patient education and monitoring rather than intervention, those with malignant potential demand histopathological confirmation, structured follow-up, and timely referral. Dental practitioners in peripheral and semi-urban settings, such as those in Namakkal and Erode, are often the first point of contact for oral health concerns and can play a central role in early detection if adequately trained in tongue lesion identification and triage.

Limitations of the study

As a single-center, cross-sectional study employing convenience sampling from one dental institution in Namakkal district, the results may not be fully generalizable to the broader or community-based population of Namakkal and Erode districts. Hospital-based convenience samples inherently overrepresent symptomatic and health-seeking individuals, potentially inflating estimated lesion prevalence.

Scope for future research

Multi-center, community-based, longitudinal studies with larger probability-sampled populations drawn from rural, peri-urban, and urban strata across Namakkal, Erode, and neighboring districts are needed to generate representative and generalizable prevalence data. Incorporating histopathological and molecular diagnostic confirmation, including cytological assessment, immunohistochemistry for lichen planus subtyping, and HPV testing for non-healing ulcers, will substantially enhance diagnostic accuracy and enable better characterization of malignant transformation risk. Comprehensive risk factor profiling, including validated habit quantification scales (type, frequency, and duration of tobacco and areca nut use), nutritional biomarkers, glycemic status, and dermatological comorbidity assessment, will permit rigorous etiological analysis and evidence-based risk stratification. Longitudinal follow-up of patients with PMDs at defined intervals is particularly important to track progression, regression, or malignant transformation and to evaluate the effectiveness of habit cessation counselling.

## Conclusions

The present study demonstrates that tongue lesions are relatively common among patients in the Namakkal and Erode districts, with predominance in middle-aged individuals and a slight male predilection. The majority of lesions were benign, with geographic tongue and fissured tongue being the most frequently observed conditions. Variations in distribution were noted across age and sex, with anemic glossitis more common in females and ankyloglossia predominantly seen in younger individuals.

Although the prevalence of PMDs was relatively low, their presence highlights the importance of routine oral examination and early detection, particularly in populations with high-risk habits such as tobacco and areca nut use. Overall, the findings emphasize the need for increased awareness, preventive strategies, and regular screening to facilitate early diagnosis and improve oral health outcomes.
